# Cobimetinib impairs tumor survival and immune escape by altering my-CAFs in BRAFV600E-mutated anaplastic thyroid carcinoma

**DOI:** 10.1186/s40164-026-00839-4

**Published:** 2026-10-20

**Authors:** Mario Udinotti, Niclas Ramm, Thamer Abed Alhaleem Amin Hamdan, Muhammad Sameer Ali Khan, Udo Siebolts, Marcus Bauer, Kerstin Lorenz, Christine Dierks, Claudia Wickenhauser, Barbara Seliger

**Affiliations:** 1 Institute of Pathology, Section Immunopathology, Martin Luther University Halle-Wittenberg, Halle, Germany; 2 Institute of Tumor Immunology, Martin Luther University Halle-Wittenberg, Halle, Germany; 3 Institute of Pathology, University Hospital, Cologne, Germany; 4 Department of Visceral, Vascular and Endocrine Surgery, Martin Luther University Halle-Wittenberg, Halle, Germany; 5 Department of Hematology/Oncology and Stem Cell Transplantation, KIM IV, Martin-Luther University Halle-Wittenberg, Halle, Germany; 6 Department of Hematology and Oncology, Freiburg University Medical Center, Albert-Ludwigs-University of Freiburg, Breisgau, Germany; 7 Institute of Translational Immunology, Center for Translational Medicine, Faculty of Health Sciences Brandenburg, Brandenburg Medical School Theodor Fontane, Brandenburg, Germany; 8 Fraunhofer Institute for Cell Therapy and Immunology (IZI), Leipzig, Germany

**Keywords:** Thyroid cancer, Tumor microenvironment, Tyrosine kinase inhibitor, Immune escape, Invasion, Cancer associated fibroblasts

## Abstract

Anaplastic thyroid carcinoma is one of the most aggressive thyroid cancers characterized by rapid progression and high resistance to conventional therapies. Although mutations in the BRAF gene are common in thyroid cancer, targeted therapies against this mutation show limited clinical benefit. This study investigated whether inhibition of downstream signaling pathways using the selected MEK inhibitor cobimetinib provides advantages over direct BRAF inhibition. Genomic and expression analyses of thyroid cancer datasets confirmed frequent BRAF alterations and activation of MAPK-pathway-associated signaling. Functional studies using thyroid cancer cell lines demonstrated that cobimetinib more effectively reduced MAPK pathway activity specifically reducing ERK1/2 phosphorylation compared to direct BRAF inhibition. This pathway suppression was associated with decreased tumor cell invasion, migration, and expression of epithelial–mesenchymal transition markers, alongside increased induction of late apoptosis. Furthermore, cobimetinib altered immune-related features of tumor cells by reducing expression of immune inhibitory molecules and cytokine secretion, while simultaneously enhancing expression of molecules involved in antigen presentation, such as HLA-ABC. Co-culture experiments with human fibroblasts showed that cobimetinib modulates tumor–fibroblast communication and fibroblast phenotype composition. Cobimetinib reduced the formation of specific immune-suppressive fibroblast subtypes, whereas exposure to interleukin-8 promoted their development and increased tumor cell migration. Secretome profiling confirmed that cobimetinib markedly depleted pro-tumorigenic cytokines, particularly reducing IL-8 secretion. While data provide preclinical evidence that downstream MEK inhibition alters tumor cell signaling and IL-8-associated tumor–fibroblast communication in ATC-centered models, they do not establish cobimetinib monotherapy as a replacement for clinically established BRAF/MEK combination therapies. Instead, these data support the broader therapeutic potential of MEK inhibitors as a strategy to target both tumor progression and immune evasion in aggressive endocrine malignancies.

**Supplementary Information:**

The online version contains supplementary material available at https://doi.org/10.1186/s40164-026-00839-4.

To the Editor,

Thyroid carcinoma (TC) represents a heterogeneous group of epithelial malignancies, ranging from well-differentiated subtypes to anaplastic thyroid carcinoma (ATC)—an aggressive form defined by rapid progression, high metastatic potential, and profound treatment resistance [1, 2]. Across thyroid cancers, genetic alterations occur in over 90% of cases, frequently involving point mutations, amplifications, and gene fusions that constitutively activate oncogenic signaling cascades [3, 4]. Among these, BRAF V600E and RAS mutations are the most prevalent, driving tumorigenesis primarily through continuous activation of the RAS–RAF–MEK–ERK pathway.

While combined dabrafenib and trametinib is the established targeted approach for BRAF V600E-mutant ATC, resistance frequently emerges via incomplete MAPK suppression driven by RAF dimerization, ERK reactivation, upstream RTK/RAS signaling, PI3K/AKT-associated bypass survival, and dedifferentiation-associated adaptations [5]. First-generation BRAF inhibitors like dabrafenib have historically shown limited clinical benefit due to paradoxical CRAF activation that sustains downstream ERK signaling. In contrast, MEK inhibitors circumvent this limitation by acting directly downstream of RAF dimerization. Furthermore, re-analysis of public genomic data supports BRAF as a dominant alteration specifically in ATC, and resistance to direct BRAF inhibition is frequently associated with impaired tumoral MHC class II antigen presentation, directly linking targeted therapy resistance to immune escape [6].

Consequently, cobimetinib was used as a mechanistic downstream MEK inhibitor to interrogate MAPK signaling, immune escape, and tumor–fibroblast communication, rather than as a proposed replacement for approved BRAF/MEK therapy. Beyond direct growth inhibition, MEK inhibition can also attenuate epithelial–mesenchymal transition (EMT) and inflammatory cytokine signaling, both influencing tumor-immune interactions [7].

## CAF heterogeneity in the tumor microenvironment

During the last decades, the immune and stromal microenvironment of TC is increasingly recognized as a determinant of tumor progression and therapy resistance. Cancer-associated fibroblasts (CAFs) constitute a dominant non-malignant population within the tumor microenvironment (TME) orchestrating extracellular matrix remodeling, cytokine secretion and immune suppression. Distinct fibroblast subtypes, such as ECM-myCAFs and TGFβ-myCAFs (Table 1), modulate T cell infiltration and immune exclusion [8–10]. In TC, CAFs have been linked to ECM remodeling, invasion, angiogenesis, and immune modulation [11], but CAF subtype definitions in ATC remain limited; therefore, the ECM-myCAF/TGFβ-myCAF marker framework was applied to interrogate stromal phenotypes in our ATC-centered system. Due to the clinical use of dabrafenib in BRAFV600E-mutant TC, its impact on oncogenic signaling, cytokine release and fibroblast reprogramming was compared with that of cobimetinib, a selective next generation MEK inhibitor with enhanced pathway suppression.

**Table 1:** Cancer-associated fibroblast (CAF) populations and subtype markers

CAF population	Subtype	Surface markers
Antigen-presenting CAFs (ap-CAFs)	—	CD86⁺, HLA-II⁺, TEM8⁻
Myofibroblastic CAFs (my-CAFs)	—	FAP⁺, TEM8⁺, α-SMA⁺, CD29High, PDGFRβ⁺
ECM-myCAFs	Subtype of CD9⁻ CAFs	CD9⁻, CD138⁺, LAMP5⁻
TGFβ-myCAFs	Subtype of CD9⁻ CAFs	CD9⁻, LAMP5⁺
IFNαβ-myCAFs	Subtype of CD9⁺ CAFs	CD9⁺, CD138⁺, LAMP5⁺
Acto-myCAFs	Subtype of CD9⁺ CAFs	CD9⁺, LAMP5⁺
Wound-myCAFs	Subtype of CD9⁺ CAFs	CD9⁺, CD138⁻, LAMP5⁻

The table classifies cancer-associated fibroblasts (CAFs) into major populations and subtypes based on their surface marker expression, highlighting the diversity (heterogeneity) of CAF types

## Cobimetinib-mediated suppression of MAPK signaling and malignant phenotypes

**Fig. 1:**
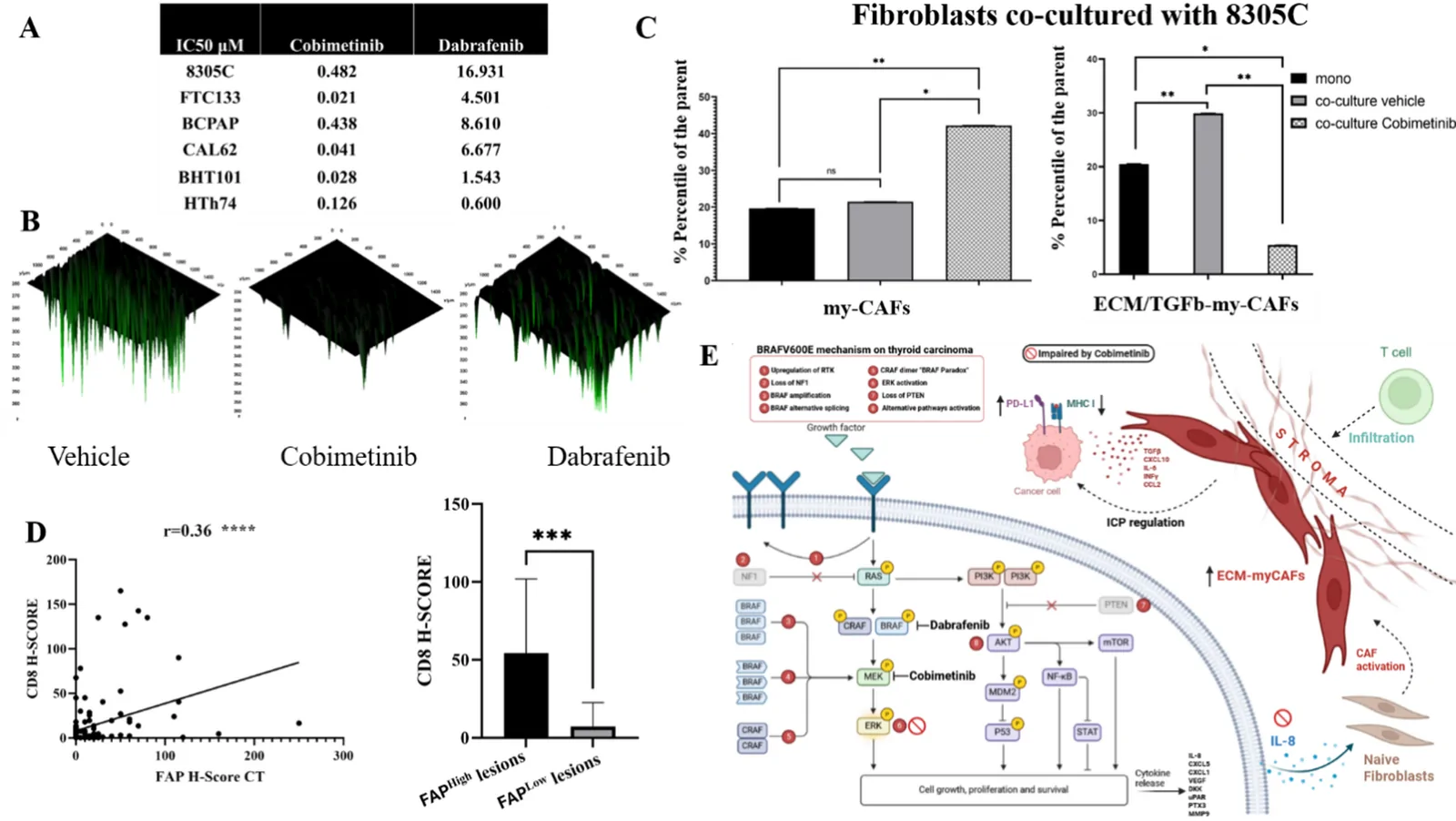
Cobimetinib suppresses tumor-intrinsic invasion and modulates CAF-associated immune phenotypes in ATC-centered thyroid carcinoma models. **A** IC50 values of Cobimetinib and Dabrafenib determined in four anaplastic thyroid carcinoma (ATC) cell lines (8305 C, CAL62, BHT101, HTh74), one follicular thyroid carcinoma cell line (FTC133), and one papillary thyroid carcinoma cell line (BCPAP). **B** Three-dimensional fluorescent projection from a gel-invasion assay’s bottom layer of 8305 C cells under vehicle, Cobimetinib, and Dabrafenib treatment, depicting relative invasive cell density. **C** Evaluation of myofibroblastic CAFs (my-CAFs) and ECM/TGFβ-my-CAFs under mono-culture, co-culture and co-culture treatment with Cobimetinib. **D** Correlation of FAP expression in connective tissues with CD8⁺ T cell infiltration in TMA lesions from the same cohort and quantification of CD8⁺ T cells in lesions stratified by high versus low/negative FAP expression. **E** Graphical abstract. Data are shown as mean ± SD. Panel C: repeated-measures one-way ANOVA with Tukey’s multiple-comparison test. Panel D: Spearman correlation for FAP/CD8 association; Mann–Whitney test for FAP-high versus FAP-low/negative lesions. *p < 0.05; **p < 0.01; ***p < 0.001; ****p < 0.0001

ATC-focused cBioPortal analyses, including GATCI Cell Reports 2024 and the MSK JCI 2016 PDTC/ATC cohort, confirmed BRAFV600E as a dominant alteration in ATC, whereas TCGA-THCA data were retained only to provide differentiated TC context (Figure  S1A). Tumor samples showed lower BRAF, but higher MEK1 and ERK2 mRNA levels with strong positive correlations between pathway components (Figure S1B-C). Cobimetinib exhibited nanomolar potency, whereas dabrafenib required micromolar concentrations to achieve IC50 values (Fig. 1A, S1D-E, S2A).

Both cobimetinib and trametinib robustly suppressed proliferation, whereas combination treatment occasionally reduced efficacy (Figure S2B-C). Across the expanded panel, cobimetinib and trametinib suppressed ERK1/2 phosphorylation more consistently than dabrafenib, which increased pERK1/2 due to the paradoxical pathway reactivation via CRAF dimerization, while AKT phosphorylation showed model-dependent compensatory changes (Figure S2D). Cobimetinib increased HLA-ABC (HLA-I), while decreasing PD-L1, IL-8, SNAI2, ZEB1 and BCL2 (Figure S2E).

Thyroid-lineage qPCR showed cobimetinib-associated induction of selected differentiation markers in 8305 C cells, accompanied by exploratory, model-dependent trends in CAL62 and BHT101 (Figure S2F). Treatment also increased late apoptosis and reduced PD-L1 surface expression on dying tumor cells (Figure S3A). Single-dose BRAF/MEK combinations served as exploratory comparators and showed combination-associated effects under the tested conditions, without formal synergy inference (Figures S3B). Furthermore, cobimetinib limited invasion and migration, with treated cells largely restricted within the gel matrix, whereas dabrafenib- and vehicle-treated cells invaded deeper layers (Fig. 1B; Figure S4A-E).

## Fibroblast reprogramming by cobimetinib

To address the poorly defined CAF biology of TC, we examined whether downstream MEK inhibition alters fibroblast phenotypes associated with antigen presentation, myofibroblastic differentiation, ECM/TGFβ programs, and IL-8-dependent tumor–stroma communication. Cobimetinib further increased HLA-ABC and CD86, but decreased PD-L1, whereas dabrafenib had minor or opposite effects, a trend mirrored by trans-well results (Figure S5A-D).

Using the Table 1 marker framework (ap-CAFs: CD86+, HLA-II+, TEM8−; my-CAFs: FAP+, TEM8+, α-SMA+, CD29High, PDGFRβ+; my-CAF subsets by CD9/CD138/LAMP5), cobimetinib reduced antigen-presenting CAFs and expanded myofibroblastic CAFs (Fig. 1C, S5E-F). Within my-CAFs, cobimetinib selectively depleted CD9 − immunosuppressive ECM-myCAFs and TGFβ-myCAFs (Fig. 1C). Supportive non-ATC TC comparator data are shown in Figure S6A–C.

## IL-8-driven tumor–stroma interactions

Proteome profiling of cobimetinib markedly reduced survivin, catalase, cIAP-1, while increasing phospho-p53 (Figure S6D). Secretome profiling demonstrated reduced IL-8/CXCL8 secretion, whereas recombinant IL-8 promoted tumor cell migration (Figure S7A–E). During prolonged IL-8 exposure, intermediate cytokine measurements supported time point-dependent modulation of CXCL10 and selected cytokines (Figure S8A); flow cytometry indicated an enrichment of the immunosuppressive CD9-/ECM-myCAF subset accompanied by increased PD-L1 expression (Figure S8B-C). Endpoint, withdrawal and IL-8 neutralization analyses point to partial, readout-dependent persistence of fibroblast phenotypes rather than stable cellular reprogramming (Figure S9).

## Therapeutic impact of MEK inhibition

In human TC tissue microarrays from untreated patients, stromal FAP/FN1 and PD-L1-related readouts correlated with CD8 + T cell infiltration (Figs. 1D, S10). Overall, the data support a preclinical model, in which downstream MEK inhibition alters tumor cell signaling and IL-8-associated tumor–fibroblast communication in ATC-focused systems. These insights require validation in established BRAF/MEK regimens and thyroid-derived fibroblast models before therapeutic conclusions can be drawn.

## Supplementary Information

Below is the link to the electronic supplementary material.

Supplementary Material 1.

Supplementary Material 2.

Supplementary Material 3.

Supplementary Material 4.

## Data Availability

All data of this study presented in the paper or the Supplementary Materials are available. All raw values are uploaded in Figshare server DOI: 10.6084/m9.figshare.28970300. They can be requested by academic researchers and will be provided after review and approval of a research proposal under Data Sharing Agreement (DSA).

## Abbreviations

AKT: Protein kinase B
ATC: Anaplastic thyroid carcinoma
BCL2: B-cell lymphoma 2
BRAFi: BRAF inhibitor
CAF: Cancer-associated fibroblast
CCL2: C-C motif chemokine ligand 2
cIAP-1: Cellular inhibitor of apoptosis protein 1
Cobimetinib: MEK inhibitor (MAP2K pathway blockade)
CRAF: RAF1 proto-oncogene serine/threonine-protein kinase
CT: Connective tissue
CXCL1: C-X-C motif chemokine ligand 1
CXCL5: C-X-C motif chemokine ligand 5
CXCL8 / IL-8: Interleukin 8
CXCL10: C-X-C motif chemokine ligand 10
Dabrafenib: BRAF inhibitor
ECM: Extracellular matrix
ECM-myCAF: Extracellular matrix-associated myofibroblastic CAF
EMT: Epithelial-to-mesenchymal transition
ERK1/2: Extracellular signal-regulated kinase ½
FAP: Fibroblast activation protein
FCS: Fetal calf serum
FN1: Fibronectin 1
HLA-ABC: Human leukocyte antigen class I complex
HRAS: Harvey rat sarcoma viral oncogene homolog
IFNαβ: Interferon alpha/beta
IL-6: Interleukin 6
IL-8: Interleukin 8
MEK: Mitogen-activated protein kinase kinase
MEKi: MEK inhibitor
NRAS: Neuroblastoma RAS viral oncogene homolog
PDGFRβ: Platelet-derived growth factor receptor beta
PD-L1 / CD274: Programmed death-ligand 1
PI3K: Phosphatidylinositol-3-kinase
PTC: Papillary thyroid carcinoma
RAS: Rat sarcoma viral oncogene family
RAF: Rapidly accelerated fibrosarcoma kinase family
rIL-8: Recombinant interleukin 8
SNAI2: Snail family transcriptional repressor 2
TC: Thyroid carcinoma
TCGA: The Cancer Genome Atlas
TGFβ: Transforming growth factor beta
TGFβ-myCAF: TGFβ-associated myofibroblastic CAF
TMA: Tissue microarray
TME: Tumor microenvironment
ZEB1: Zinc finger E-box-binding homeobox 1
