# Supplementary material for: Cobimetinib impairs tumor survival and immune escape by altering my-CAFs in BRAFV600E-mutated anaplastic thyroid carcinoma
Source: Exp Hematol Oncol. 2026 Oct 20;15:107. doi: 10.1186/s40164-026-00839-4 (PMC13681933; doi:10.1186/s40164-026-00839-4)
Supplement: Supplementary file 1 [file 40164_2026_839_MOESM1_ESM.docx]

**Table S1**

| **Material** | **Company** | **Cat.Number** |
| --- | --- | --- |
| Trametinib | SelleckChem | S2673 |
| Cobimetinib | SelleckChem | S8041 |
| Dabrafenib | SelleckChem | S2807 |
| mouse zenon 594 | Invitrogen | Z25007 |
| Phospho-p44/42 MAPK (Erk1/2) (Thr202/Tyr204) | CellSignaling | 9101 |
| Phospho-Akt (Ser473) (D9E) XP^®^ rabbit mAb | CellSignaling | 4060 |
| p44/42 MAPK (Erk1/2) (137F5) Rabbit Monoclonal Antibody | CellSignaling | 4695 |
| Akt (pan) (11E7) Rabbit Monoclonal Antibody | CellSignaling | 4685 |
| GAPDH (14C10) rabbit mAb | CellSignaling | 2118 |
| Rabbit anti-mouse IgG (HRP conjugate) | CellSignaling | 58802 |
| Mouse anti-rabbit IgG (HRP conjugate) | CellSignaling | 93702 |
| TEM8 405 | Novus | 5658AF 405 |
| HLA ABC APC | Biolegend | 311410 |
| HLA DP/DQ/DR APC cy7 | Novus | 64358 APC cy7 |
| PDL1 PE | Biolegend | 124808 |
| CD9 APC cy7 | Abcam | AB239306 |
| CD86 PE/Dazzle | Biolegend | 374218 |
| aSMA APC | RnD | IC1420A |
| CD29 PE | RnD | FAB17781F |
| CD29 BV510 | BD bioscience | 563514 |
| FAP-unconjugated-mouse | RnD | MAB3715 |
| CD138 FITC | Biolegend | 352303 |
| LAMP5 PE | Miltenyi Biotec | 130-109-203 |
| PDGFRb alexa790 | Biocompare | sc-373805 AF790 |
| FITC mouse IgG1, κ isotype | Biolegend | 400109 |
| Brilliant violet 510™ mouse IgG1 | Biolegend | 400171 |
| APC mouse IgG2a, κ isotype | Biolegend | 400219 |
| Mouse IgG_1_ alexa Fluor® 405-conjugated Antibody | RnD | IC002V |
| APC-Cy™7 mouse IgG2a, κ isotype | BD bioscience | 557751 |
| PE mouse IgG1, κ isotype | Biolegend | 400111 |
| PE/Dazzle™ 594 mouse IgG1, κ isotype | Biolegend | 981814 |
| FITC annexin V apoptosis detection kit with PI | Biolegend | 640914 |
| Crystal violet solution | Merck | 548-62-9 |
| VitroGel® 3D high concentration | TheWell Bioscience | TWG001 |
| Proteome profiler human apoptosis array kit | RnD | ARY009 |
| Invitrogen bolt 4%-12% Bis Tris Plus western blot gels | Invitrogen | 22122010 |
| Taq Pro universal SYBR qPCR master mix | VAZYME | Q712-03 |
| SignalFire^™^ ECL reagent | CellSignaling | 6883 |
| VitroGel® hydrogel matrix | TheWell bioscience | VHM01 |
| Page ruler western blot ladder | Thermo scientific | 1194444 |
| BioRAD CFXConnect qPCR | BioRAD |  |
| iBright 750 | Invitrogen | A44116 |
| iBlot PVDF | Invitrogen | IB24002 |
| iBlot nitrocellulose | Invitrogen | IB25002 |
| iBlot2 machine | Invitrogen | IB21001 |
| TECAN Infinite M200 Pro | Tecan |  |
| EDTA protease / phosphatase 100x | Thermo scientific | 78440 |
| Bovine serum albumin fraction V | Sigma-Aldrich | 10735094001 |
| Intracellular fixation & permeabilization buffer set | Thermo scientific | 88-8824-00 |
| Agarose | Serva | 9012-36-6 |
| RevertAid first strand cDNA synthesis kit | Thermo Fisher | k1622 |
| LEGENDplex™ HU essential immune response panel | Biolegend | 740930 |
| Intracellular transcription factor buffer kit | BD Pharmigen | 562574 |
| Gibco RPMI 1640X | Gibco | 29875-034 |
| Gibco Opti med | Gibco | 31985-047 |
| Lonza EMEM | Lonza | 12-125F |
| FETAL+ | Anprotec | AC-5M-0161 |
| L-Glutamine | Sigma-Aldrich | G7513 |
| Pen/Strep antibiotics | Sigma-Aldrich | P0781 |
| Trypsine | Gibco | 15400-054 |
| Incubator BBD6220 | Thermo scientific |  |
| Hood HERA SAFE | Kendro |  |
| 12-well transwell insert 0.4μm | Qart | 335870 |
| 24-well transwell insert 8μm | CORNING | 3464 |
| RNA isolation kit | Machery-Nagel | 740955 250 |
| PIERCE BCA Protein kit | Thermo scientific | 23225 |
| Nucleo bond plasmid purification kit | Machery-Nagel | 74041 250 |
| Proteome profiler cytokine XL | RnD | ARY022B |
| Proteome profiler apoptosis | RnD | ARY009 |
| Culture-Insert 4 Well in µ-Dish 35 mm | Ibidi | 80466 |
| Recombinant IL-8 | Biolegend | 574202 \| |
| Human IL-8/CXCL8 antibody | RnD | MAB208 |
| Human CXCR1/IL-8RA antibody | RnD | MAB330 |

| **PRIMER TARGET** | **Sequence** |
| --- | --- |
| PDL1 |  |
| Forward primer | 5΄-TGCCGACTACAAGCGAATTACTG-3΄ |
| Reverse primer | 3΄-CTGCTTGTCCAGATGACTTCGG-5΄ |
| HLA-A |  |
| Forward primer | 5΄-AGATACACCTGCCATGTGCAGC-3΄ |
| Reverse primer | 3΄-GATCACAGCTCCAAGGAGAACC-5΄ |
| HLA-B |  |
| Forward primer | 5΄-CTGCTGTGATGTGTAGGAGGAAG-3΄ |
| Reverse primer | 3΄-GCTGTGAGAGACACATCAGAGC-5΄ |
| HLA-C |  |
| Forward primer | 5΄-GGAGACACAGAAGTACAAGCGC-3΄ |
| Reverse primer | 3΄-ACATCCTCTGGAGGGTGTGAGA-3΄ |
| B2M |  |
| Forward primer | 5΄-CCACTGAAAAAGATGAGTATGCCT-3΄ |
| Reverse primer | 3΄-CCAATCCAAATGCGGCATCTTCA-5΄ |
| VIM |  |
| Forward primer | 5΄-AGGCAAAGCAGGAGTCCACTGA-3΄ |
| Reverse primer | 3΄-ATCTGGCGTTCCAGGGACTCAT-5΄ |
| FN1 |  |
| Forward primer | 5΄-ACAACACCGAGGTGACTGAGAC-3΄ |
| Reverse primer | 3΄-GGACACAACGATGCTTCCTGAG-5΄ |
| SNAI2 |  |
| Forward primer | 5΄-CCCTCACTGCAACAGAGCAT-3΄ |
| Reverse primer | 3΄-TGCGTCACTCAGTGTGCTAC-5΄ |
| ZEB1 |  |
| Forward primer | 5΄-GGCATACACCTACTCAACTACGG-3΄ |
| Reverse primer | 3΄-TGGGCGGTGTAGAATCAGAGTC-5΄ |
| ACTA2/aSMA |  |
| Forward primer | 5΄-CTATGCCTCTGGACGCACAACT-3΄ |
| Reverse primer | 3΄-CAGATCCAGACGCATGATGGCA-5΄ |
| PDGFRA |  |
| Forward primer | 5΄-GACTTTCGCCAAAGTGGAGGAG-3΄ |
| Reverse primer | 3΄-AGCCACCGTGAGTTCAGAACGC-5΄ |
| FAP |  |
| Forward primer | 5΄-GGAAGTGCCTGTTCCAGCAATG-3΄ |
| Reverse primer | 3΄-TGTCTGCCAGTCTTCCCTGAAG-5΄ |
| BCL2 |  |
| Forward primer | 5΄-ATCGCCCTGTGGATGACTGAGT-3΄ |
| Reverse primer | 3΄-GCCAGGAGAAATCAAACAGAGGC-5΄ |
| BAX |  |
| Forward primer | 5΄-TCAGGATGCGTCCACCAAGAAG-3΄ |
| Reverse primer | 3΄-TGTGTCCACGGCGGCAATCATC-5΄ |
| BRAF |  |
| Forward primer | 5΄-AACGAGACCGATCCTCATCAGC-3΄ |
| Reverse primer | 3΄-GGTAGCAGACAAACCTGTGGTTG-5΄ |
| IL-8 |  |
| Forward primer | 5΄-GAGAGTGATTGAGAGTGGACCAC-3΄ |
| Reverse primer | 3΄-CACAACCCTCTGCACCCAGTTT-5΄ |
| SLC5A5 (NIS) |  |
| Forward primer | 5΄-CTCTGCTGGTGCTGGACATCTT-3΄ |
| Reverse primer | 3΄-GAGGTCTTCTACAGTGACTGCAG-5΄ |
| TG |  |
| Forward primer | 5΄-CCAGTGGCTTCTCTTCCTGACT-3΄ |
| Reverse primer | 3΄-CCTTGGAGGAAGCGGATGGTTT-5΄ |
| TSHR |  |
| Forward primer | 5΄-GAGTTTCCTTCACCTCACACGG-3΄ |
| Reverse primer | 3΄-CTGCTCTCATTACACATCAAGGAC-5΄ |
| TPO |  |
| Forward primer | 5΄-CACCAGGCTTTCTTCAGCCCAT-3΄ |
| Reverse primer | 3΄-GGACAGCACAAAGAGCCTTTCC-5΄ |
| PAX8 |  |
| Forward primer | 5΄-TCAACCTCCCTATGGACAGCTG-3΄ |
| Reverse primer | 3΄-GAGCCCATTGATGGAGTAGGTG-5΄ |
