# Supplementary material for: Cobimetinib impairs tumor survival and immune escape by altering my-CAFs in BRAFV600E-mutated anaplastic thyroid carcinoma
Source: Exp Hematol Oncol. 2026 Oct 20;15:107. doi: 10.1186/s40164-026-00839-4 (PMC13681933; doi:10.1186/s40164-026-00839-4)
Supplement: Supplementary file 2 [file 40164_2026_839_MOESM2_ESM.docx]

**Table S2.** Cell line identity verification by STR profiling

| **Cell line** | **Model / classification in manuscript** | **STR provider** | **Report ID** | **Report date** | **STR reference match** | **STR conclusion** | **Cellosaurus reference noted in report** | **Mycoplasma status** | **Notes** |
| --- | --- | --- | --- | --- | --- | --- | --- | --- | --- |
| 8305C | ATC model | Microsynth AG | 03269_019786 | 09.07.2026 | 100% | Matched reference profile; no detectable human-origin contamination. | CVCL_1053 | Negative by routine in-house PCR | Authenticated. |
| CAL62 | ATC model | Microsynth AG | 03269_019787 | 09.07.2026 | 100% | Matched reference profile; no detectable human-origin contamination. | CVCL_1112 | Negative by routine in-house PCR | Authenticated. |
| BHT101 | ATC model | Microsynth AG | 03269_019788 | 09.07.2026 | 91.8% | Matched reference profile; no detectable human-origin contamination. | CVCL_1085 | Negative by routine in-house PCR | Authenticated with lower concordance than 100%; interpret transparently. |
| HTh74 | ATC model | Microsynth AG | 03269_019789 | 09.07.2026 | 80% | Lower match; report states contamination with human origin. | CVCL_6288 | Negative by routine in-house PCR | Authenticated with lower concordance than 100%; interpret transparently. |
| FTC133 | Follicular thyroid carcinoma comparator | Microsynth AG | 03269_019790 | 09.07.2026 | 100% | Matched reference profile; no detectable human-origin contamination. | CVCL_1219 | Negative by routine in-house PCR | Authenticated. |
| BCPAP | Non-ATC thyroid carcinoma comparator (Now considered as PDTC) | Microsynth AG | 03269_019793 | 09.07.2026 | 98.4% | Matched reference profile; no detectable human-origin contamination. | CVCL_0153 | Negative by routine in-house PCR | Authenticated. |
| Nthy-ori 3-1 | Non-malignant thyroid epithelial comparator | Microsynth AG | 03269_019791 | 09.07.2026 | 100% | Matched reference profile; no detectable human-origin contamination. | CVCL_2659 | Negative by routine in-house PCR | Authenticated. |
| BJ fibroblasts | Standardized fibroblast model | Microsynth AG | 03269_019792 | 09.07.2026 | 100% | Matched reference profile; no detectable human-origin contamination. | CVCL_3653 | Negative by routine in-house PCR | Authenticated. |
