# Supplementary material for: Cobimetinib impairs tumor survival and immune escape by altering my-CAFs in BRAFV600E-mutated anaplastic thyroid carcinoma
Source: Exp Hematol Oncol. 2026 Oct 20;15:107. doi: 10.1186/s40164-026-00839-4 (PMC13681933; doi:10.1186/s40164-026-00839-4)
Supplement: Supplementary file 3 [file 40164_2026_839_MOESM3_ESM.docx]

**Materials and Methods**

All material and plastic ware used in this study can be found on Table S1 along with catalog numbers and information of the companies.

Cell lines, cell culture and treatment

The revised experimental validation focused on the cell panel comprised by ATC cell lines 8505C, 8305C, CAL62, BHT101, and HTh74; the follicular thyroid carcinoma model FTC133; the non-ATC thyroid carcinoma model BCPAP Previously considered as PTC and now is considered as PDTC); the immortalized non-malignant thyroid epithelial line Nthy-ori 3-1; and BJ fibroblasts. Supportive data from the ATC models, HTh74 and 8505C where applicable, are not used as standalone evidence for the conclusions. Because the manuscript is ATC-centered, FTC133 and BCPAP are presented as supportive thyroid carcinoma comparators rather than as core ATC models. These cell lines kindly provided by S. Huettelmaier (Martin Luther University Halle-Wittenberg, Institute of Molecular Medicine, Halle, Germany) were maintained in RPMI-1640 medium supplemented with 10% fetal calf serum (FCS), (Anprotec, Bruckberg, Germany) glutamine and 1% penicillin-streptomycin (Sigma Aldrich, Germany). Normal thyroid epithelial cells (Nthy-ori-3-1) and human foreskin naïve fibroblasts (BJ) were cultured in EMEM medium supplemented with 10% FCS (Anprotec), glutamine and 1% penicillin-streptomycin (Sigma Aldrich, Germany). All cell lines were incubated at 37°C in a humidified atmosphere containing 5% CO₂. For analyses of the effects of inhibitors on the TC phenotype and growth characteristics, TC cells were either left untreated or treated with the MEKi Cobimetinib and the BRAFi Dabrafenib (SelleckChem, Germany). Initial dose-response experiments were performed using 0.01–10 µM inhibitor concentrations for 48 h to determine IC50 values. For mechanistic and phenotypic assays, including qPCR, immunoblotting, migration, invasion and flow-cytometric analyses, cells were treated for 48 h with 1 µM Cobimetinib, Dabrafenib or Trametinib unless otherwise stated. This standardized concentration was selected to allow direct comparison between inhibitors, cell lines and readouts within the tested dose range. BJ fibroblasts were cultured in the absence or presence of 10 ng/mL rIL8 (Biolegend, Germany) for 1 to 8 weeks. For analysis of the reversibility of the IL-8-mediated changes, BJ cells were treated for 4 weeks with rIL-8 followed by their culturing in complete medium for additional 4 weeks prior to analysis. All materials can be found on Table S1 along with catalog numbers and companies.

STR and mycoplasma

Cell-line identity was verified by STR profiling performed by Microsynth AG on 09.07.2026 using the PowerPlex® 16 HS System, ABI3730xl fragment analysis and GeneMarker HID software. STR profiles were compared with Cellosaurus reference profiles and are summarized in Table S2. All cell lines were routinely tested for mycoplasma contamination by in-house PCR and were negative during the revision period.

Determination of the cell viability and migration

For analysis of cell viability, cells were seeded in 96-well plates and treated for 48 hours with vehicle, single agents or drug combinations, respectively. Following treatment, cells were fixed with 4% paraformaldehyde and stained with 0.5% crystal violet (Merck, Germany) for 30 minutes. After removing the excess stain with distilled water and drying, crystal violet was solubilized with 0.1% SDS and absorbance was measured at 570 nm using a microplate reader.

For migration assays, cells were seeded in serum-free medium in the upper chambers of 8 μm pore trans-well inserts. The lower chamber contained complete medium supplemented with 10% FCS as a chemoattractant. After 24 hours, non-migrated cells were removed from the upper surface and cells migrating to the lower chamber were fixed, stained with 0.5% crystal violet and then quantified by solubilizing the dye and reading absorbance.

Invasion assay and confocal Z-stack imaging

Invasion assays were performed by embedding carboxyfluorescein diacetate succinimidyl ester (CFSE)-labeled TC cells in a 1:1 mixture of Vitrogel 3D hydrogel (Thewell bioscience, USA) and culture medium. 100 μL of the cell-hydrogel suspension/well was seeded using a black, clear-bottom 96-well plate. After polymerization at room temperature, an additional 100 μL of complete medium containing 10% FCS was gently added. Plates were incubated for 48 hours at 37°C in a humidified incubator with 5% CO₂ to allow cell invasion through the gel.

Following incubation, Z-stack images were acquired using a laser scanning confocal microscope at 5 μm intervals, capturing the full vertical range of the gel matrix from top to bottom and analyzed using ImageJ (Fiji distribution). Invasion depth interpolation was carried out using the “Plot Z-axis Profile” function. A maximum intensity projection was created using the “Z Project” function, and the “3D Viewer” plugin was used for 3D rendering and visualization of the invasion pattern.

To quantify the number of cells that reached the bottom of the gel, the final confocal slice (representing the well bottom) was extracted from the Z-stack. This image was converted to grayscale if necessary, and background fluorescence was reduced using the “Subtract Background” tool. A region of interest (ROI) was manually defined to cover the entire bottom surface of the well. The mean fluorescence intensity (MFI) within the ROI was then measured using the “Measure” function in ImageJ, yielding a relative metric of CFSE-labeled cells that had completed the invasion process.

Wound healing assay

Wound healing assays were performed as recently described(29, 30). Briefly, cells were grown to confluence in wound healing plates (Ibidi, Germany). A uniform scratch was created using the removable silicon chamber after confluence followed by the addition of vehicle or rIL8. Wound closure was imaged at 0 and 48 hours and the wound area was quantified using Biostation software and data normalized to baseline.

Quantitative RT-PCR

Total cellular RNA was isolated using the RNA Isolation Kit (Machery & Nagel, Germany) following the manufacturer’s protocol. Complementary DNA (cDNA) was synthesized from 0.5 µg total RNA using the ReverseAid First Strand cDNA Synthesis Kit (Thermo Fisher, Germany) according to the manufacturer’s instructions. Quantitative real-time PCR (qPCR) was performed using the SYBR Green PCR Master Mix on a Bio-Rad CFX96 Touch Real-Time PCR Detection System. The relative expression levels of target genes were normalized to housekeeping gene controls using the ΔΔCt method and are presented as log₂ fold changes relative to control conditions. All primer sequences used in this study are listed in Supplementary (Table S1). For thyroid EMT and Immunomodulatory marker analysis, qPCR was performed for HLA-ABC, B2M, PD-L1, Il-8, BRAF, BAX, BCL2, FAP, FN1, VIM, PDGFRa, SNAI2, ZEB1, ACTA2 after 48 h treatment with vehicle, Dabrafenib, Cobimetinib and for combinatory treatment. For thyroid-lineage marker analysis, qPCR was performed for SLC5A5/NIS, TG, TPO, TSHR and PAX8 after 48 h treatment with vehicle, Dabrafenib, Cobimetinib or Trametinib. 8305C experiments were performed as n=3 biological replicates and statistically analyzed using ΔCt values; CAL62 and BHT101 were included as exploratory single-experiment validation where indicated.

Western blot analysis

Total cellular proteins were extracted using RIPA buffer supplemented with protease and phosphatase inhibitors. Protein concentrations were determined using the Pierce BCA Protein Assay Kit. 10–30 µg protein/lane were subjected to SDS-PAGE and transferred to either polyvinylidene difluoride (PVDF) or nitrocellulose (NC) membranes (Thermo Fisher, Germany), respectively, using the iBlot2 Dry Blotting System. Membranes were blocked with 5% bovine serum albumin (BSA) in TBS-Tween (TBS-T) for 1 hour at room temperature. The membranes were incubated with primary antibodies (Abs) overnight at 4°C followed by incubation with HRP-conjugated secondary Abs for 1 hour at room temperature. Signal detection was carried out using an enhanced chemiluminescence (ECL) system and membranes were imaged using the iBright FL750 Imaging System.To assess the expression of secreted protein factors, proteome profiler cytokine arrays were used. Proteome arrays were processed according to the manufacturer’s instructions. Signal intensities were quantified using the iBright FL750 Imaging System and normalized as described in the respective Figure legends. For expanded MAPK/PI3K signaling analysis, cells were treated for 48 h with vehicle, 1 µM Dabrafenib, 1 µM Cobimetinib or 1 µM Trametinib. Immunoblots were probed for pERK1/2, total ERK1/2, pAKT, total AKT and GAPDH.

Flow cytometry

Flow cytometry was performed as recently described (31). For staining of membranous markers, cells were harvested with trypsin, washed in FACS buffer (PBS + 2% FCS) and incubated with fluorescence conjugated Abs directed against surface markers (e.g., CD274, HLA-ABC, CD80, CD86) for 30 minutes at 4°C. After washing, the cells were analyzed on a CytoFLEX LX instrument (Beckman Coulter). For intracellular staining of markers, cells were fixed in fixation buffer for 20 minutes at room temperature before the Ab (anti-LAMP5, anti-α-SMA) was added for 30–45 minutes at 4°C according to the manufacturers’ protocol. Cells were washed in the permeabilization buffer, resuspended in FACS buffer and immediately analyzed by flow cytometry.

Cytokine analysis was performed using the LEGENDplex™ Human Inflammation Panel following the manufacturer’s instructions. Cell culture supernatants were incubated with fluorescent bead-based capture antibodies, followed by PE-conjugated detection reagents. Samples were acquired on a CytoFLEX LX flow cytometer (Beckman Coulter) and data analyzed with the FlowJo™ software using standard curves generated by five-parameter logistic regression.

For determination of apoptosis, cells were stained with annexin V-FITC and propidium iodide (PI). After staining, samples were analyzed for early and late apoptosis by flow cytometry. PD-L1 surface expression on Annexin V/PI-gated subpopulations was also assessed.

Co-culture experiments with fibroblasts and characterization of CAF subpopulations
For indirect co-culture, BJ fibroblasts were seeded in the upper chamber of a 0.4μm trans-well system, while 8305C cells were seeded in the lower chamber. For direct co-culture, BJ fibroblasts were pre-labeled with CFSE and co-seeded with unlabeled 8305C cells. After 48 hours, CFSE⁺ fibroblasts and CFSE⁻ cancer cells were separately gated and analyzed by flow cytometry for the expression of immune checkpoint (ICP) molecules, components of the antigen presentation machinery (APM) and markers for CAF subpopulations. In addition, co-cultures were treated with the MEKi Cobimetinib at a concentration range between 0.01–10 μM, with 0.4 μg/mL of anti-IL8 or anti-CXCR1 (RnD, Germany) for 48 hours, respectively, before the cells were subjected to flow cytometry as described above.

Immunohistochemical analysis of TC specimen

Conventional IHC was conducted using tissue microarrays (TMA) comprising of 187 formalin-fixed paraffin-embedded (FFPE) primary TC samples representing distinct histological subtypes. The use of these samples was approved by the Ethical Committee of the Medical Faculty, Martin Luther University Halle-Wittenberg, Germany (approval number 2017–81). All TC specimens of the TMA were independently evaluated by two pathologists (U. Siebolts and M. Bauer) blinded to the clinical data. Immunohistochemical staining was performed using primary Abs directed against the fibroblast activation protein (FAP), CD274/PD-L1, CD8 and fibronectin-1 (FN1), respectively.

Bioinformatics and statistical analysis

In silico analyses of The Cancer Genome Atlas (TCGA) datasets were performed of a cohort of 492 TC cases comprising primary and metastatic tumors predominantly of PTC, Anaplastic Thyroid Cancers (GATCI, Cell Reports 2024) (https://www.cbioportal.org) 190 ATC samples and Poorly-Differentiated and Anaplastic Thyroid Cancers (MSK, JCI 2016) 84 PDTC+ 33 ATC samples, to determine the mutational burden. The Gene Expression Profiling Interactive Analysis (GEPIA) database was employed to compare both RNA and protein expression levels of selected targets between 512 TC samples and 337 normal thyroid tissues. Pearson correlations between selected targets within the same cohort were performed to determine linear relationships between continuous variables.

Drug sensitivity of tumor cell lines was analyzed in silico using the Genomics of Drug Sensitivity in Cancer (GDSC) database. Pearson correlation matrices for IC50 values of chemotherapy drugs across 918 cancer cell lines were generated, including 16 TC cell lines as well as comparative IC50 analyses between wild type and BRAF-mutated TC cell lines. Statistical analyses were selected according to the structure of each experiment. Unless otherwise stated, in-house experiments were performed as n = 3 independent biological replicates in a paired design, in which each replicate was split across all treatment conditions. Two paired conditions were analyzed using paired two-tailed t-tests. More than two paired conditions were analyzed using repeated-measures one-way ANOVA followed by Tukey’s multiple-comparison test when all pairwise comparisons were assessed. Independent tissue-group comparisons were analyzed using Mann–Whitney tests. Correlation analyses were performed using Spearman correlation unless otherwise specified. Normality was assessed using the Shapiro–Wilk test where sample size permitted; for small exploratory datasets or non-normally distributed data, non-parametric analyses or descriptive reporting were used as indicated. Data are shown as mean ± SD unless otherwise indicated. Statistical significance was defined as follows: *p < 0.05, **p < 0.01, ***p < 0.001, ****p < 0.0001.
