# Supplementary material for: Cobimetinib impairs tumor survival and immune escape by altering my-CAFs in BRAFV600E-mutated anaplastic thyroid carcinoma
Source: Exp Hematol Oncol. 2026 Oct 20;15:107. doi: 10.1186/s40164-026-00839-4 (PMC13681933; doi:10.1186/s40164-026-00839-4)
Supplement: Supplementary file 4 [file 40164_2026_839_MOESM4_ESM.docx]

Figure S1. Genomic and pharmacologic context of MAPK pathway targeting in ATC-centered thyroid carcinoma models.

(A) Mutational landscape of papillary thyroid carcinoma samples derived from The Cancer Genome Atlas (TCGA) (n=492), two anaplastic thyroid carcinoma sample cohorts from (GATCI, Cell Reports 2024) (MSK, JCI 2016) (n=223) and five of our model cell lines from Cell Model Passports, highlighting the frequency of BRAFV600E among other genetic alterations, along with the mutation type and percentile in patients. (B) RNA expression levels in TC tissues (n = 512) versus normal thyroid tissues (n = 337) analyzed using the GEPIA (Gene Expression Profiling Interactive Analysis) database. (C) Protein expression correlations within thyroid tumor samples based on proteomic data. (D) Correlation analysis of MEK inhibitors with the BRAF inhibitor Dabrafenib using the Genomics of Drug Sensitivity in Cancer (GDSC) database. (E) Comparative analysis of effective concentration (IC50) values for Dabrafenib and Trametinib (a MEK inhibitor) in BRAF-mutant versus BRAF wild-type cell lines, illustrating mutation-specific drug responsiveness. Public-dataset analyses were performed using the statistical workflows of the corresponding online platforms and are presented as exploratory. Pearson correlation was predefined by the website for panel C and Spearman correlation was also predefined from the website for panel D.

Figure S2. MEK inhibition alters viability, MAPK/AKT signaling, immune-escape markers, EMT markers and thyroid-lineage markers.

(A) Cell growth values of two TC cell lines with Cobimetinib and Dabrafenib at different concentrations (n=3). (B) Proliferation assay performed in six TC cell lines treated with equimolar concentrations of Dabrafenib, Cobimetinib, and a combination of both drugs (n=3). (C) Viability assay with Crystal violet stain performed in five TC cell lines and one normal thyroid cell line treated with equimolar concentrations of Dabrafenib, Cobimetinib and Trametinib (n=3) and their respective image pictures after 48h. (D) Representative Immunoblot analysis of phosphorylated downstream targets (e.g., pERK1/2) following treatment with Cobimetinib, Dabrafenib and Trametinib in multiple thyroid carcinoma cell lines and one normal thyroid cell line. (E) Quantitative PCR (qPCR) analysis of 8305C cells for the expression of immune checkpoint molecules, antigen-presenting molecules and EMT markers following treatment with Cobimetinib, Dabrafenib and co-treatment, presented as relative ratio from the vehicle in log2. (F) qPCR on thyroid differentiation markers between Cobimetinib , Dabrafenib and Trametinib on 8305C (n=3) and CAL62 (n=1), BHT101 (n=1). Data are mean ± SD from n=3 biological replicates where indicated. Dose-response curves were fitted by nonlinear regression to estimate IC50 values. Paired multi-condition comparisons were analyzed by repeated-measures one-way ANOVA with Tukey’s test. Differentiation qPCR was statistically analyzed for 8305C only; CAL62 and BHT101 are shown as descriptive single-experiment validation. *p < 0.05; **p < 0.01; ***p < 0.001

Figure S3. MEK inhibition increases apoptotic cell fractions and modulates PD-L1 expression on apoptotic tumor cells.

(A) Annexin V/PI apoptosis assay in 8305C, BCPAP and Nthy-ori 3-1 (normal thyroid epithelial) cells assessing the apoptotic stage distribution and PD-L1 surface expression across apoptotic subpopulations (n=3), representative flow-cytometry plots are shown for 8305C and are directly labelled. (B) Expanded Annexin V/PI apoptosis assay in multiple thyroid carcinoma cell lines and 3 different individual and combinatory treatments assessing viability and PD-L1 surface expression. Data are presented as means and were analyzed by two tail paired t-test for panel A. Single-experiment exploratory data on panel B are shown descriptively without statistical testing. *p < 0.05; **p < 0.01; ***p < 0.001

Figure S4. Cobimetinib reduces invasion and migration of thyroid carcinoma cells.

(A) Schematic presentation of the invasion assay workflow using CFSE-stained cells embedded in Vitrogel. Z-stack images (5 μm intervals) were acquired after 48 hours for total invasion reconstruction. Fluorescence at the bottom of the well was captured to quantify penetrated cells. (B) Z-stack image reconstruction and interpolated invasion depth of 8305C cells under vehicle, Cobimetinib and Dabrafenib treatment. (C) Quantitative analysis of mean fluorescence intensity at the bottom of the well from two TC cell lines across the three treatment conditions (n=3). (D) Schematic illustration of the migration assay setup with cells migrating through a trans-well insert (no FCS in upper chamber) towards a FCS-containing lower chamber under the same three treatment conditions. (E) Migration assay results from five TC cell lines and one normal thyroid cell line quantified by crystal violet staining of migrated cells at the bottom of the well and statistical comparison of mean cell migration (n=3). Data are shown as mean ± SD from n=3 biological replicates. Invasion and migration were analyzed within each cell line using repeated-measures one-way ANOVA followed by Tukey’s multiple-comparison test. *p < 0.05; **p < 0.01; ***p < 0.001

Figure S5. Cobimetinib modulates immune-checkpoint and antigen-presentation markers in tumor–fibroblast co-culture systems.

(A)Trans-well-based indirect co-culture of naïve BJ fibroblasts with 8305C cells analyzing fibroblast surface expression of immune checkpoint and antigen-presenting molecules under different treatment conditions, depicted in log2 ratio compared to mono-culture. (B-C) Direct co-culture of BJ fibroblasts (CFSE⁺) and 8305C cells (CFSE⁻), simultaneously analyzed for the same markers in both cell types across treatment conditions, depicted in log2 ratio compared to vehicle. (D) FAP^+^ , TEM8^-^ Fibroblasts subset co-cultured with 2 different TC cell lines and one normal thyroid cell line and analyzed for immune checkpoint and antigen-presenting molecules under different treatment conditions, depicted in log2 ratio compared to mono-culture. (E-F) Flow cytometric assessment of antigen-presenting CAFs (ap-CAFs) under mono-culture, co-culture and co-culture with Cobimetinib conditions. Data are mean ± SD from n=3 biological replicates. Marker expression was analyzed separately for each marker using repeated-measures one-way ANOVA with Tukey’s test, or paired two-tailed t-test for two-condition comparisons. Direct co-culture values are interpreted relative to vehicle-treated co-cultures, single-experiment exploratory data are shown descriptively without statistical testing. . *p < 0.05; **p < 0.01; ***p < 0.001

Figure S6. Cobimetinib alters CAF-subpopulation distribution and apoptosis-related signaling in supportive thyroid carcinoma models.

(A) Flow cytometric histoplot and dot plot analysis of antigen-presenting CAFs (ap-CAFs) in BJ fibroblasts under mono-culture, co-culture with BCPAP cells and the absence and presence of Cobimetinib. (B) Same experimental setup as in (A) analyzing the myofibroblastic CAF (my-CAF) population. (C) Analysis of ECM/TGFβ-associated myofibroblastic CAFs (ECM/TGFβ-myCAFs) in BJ fibroblasts under mono-culture, BCPAP co-culture, and BCPAP co-culture with Cobimetinib conditions, as directly annotated in the panel. (D) Proteome profiler array of apoptosis-related markers in 8305C total protein lysate treated with vehicle or Cobimetinib, categorized using the STRING database. CAF-population frequencies were analyzed using repeated-measures one-way ANOVA followed by Tukey’s test. Proteome-profiler array data are shown descriptively unless replicate quantification is indicated. *p < 0.05; **p < 0.01; ***p < 0.001

Figure S7 Cobimetinib reduces IL-8 secretion and IL-8 promotes tumor-cell migration.

(A) Proteome profiler array detecting 105 cytokines in the 8305C cell line supernatant after 48 hours of vehicle or Cobimetinib treatment; cytokines categorized using the STRING database. (B) Selected panel depiction of core immune-related cytokines from 8305C supernatant comparing vehicle and Cobimetinib treatment. (C) Quantitative analysis of IL-8 secretion from the same panel across five TC cell lines and one normal TC cell line under Cobimetinib treatment. (D) Representative wound closure images of recombinant IL-8–treated TC cells after 48 hours with wound boundaries outlined. (E) Statistical comparison of wound closure of 8305C and BCPAP cell lines following IL-8 stimulation. Data are mean ± SD from n=3 biological replicates where indicated. IL-8 secretion was analyzed within each cell line using paired two-tailed t-tests with Holm–Šídák correction for multiple cell-line comparisons. Wound closure was analyzed within each cell line using paired two-tailed tests with correction where applicable. *p < 0.05; **p < 0.01

Figure S8 Prolonged recombinant IL-8 exposure induces time-point-dependent cytokine changes and partial fibroblast phenotype persistence.

(A) Two-month treatment of naïve BJ fibroblasts with recombinant IL-8 followed by assessment of 13-plex human immune response cytokine panel. Flow cytometry histograms showing CXCL10 expression after 8 weeks. Statistical analysis of CXCL10, free active TGFβ1, IL-4, IFNγ and CCL2 levels at week 1 and week 8 comparing IL-8–treated and untreated fibroblasts. (B) Flow cytometric quantification of ECM/TGFβ myofibroblasts (CD9⁻) and wound/acto/IFNαβ myofibroblasts (CD9⁺) in control fibroblasts, fibroblasts treated with IL-8 for 8 weeks and fibroblasts exposed to recombinant IL-8 for 4 weeks followed by culture in complete medium without recombinant IL-8 for an additional 4 weeks. (C) Surface PD-L1 expression on total BJ fibroblasts comparing untreated and 8-week IL-8–treated conditions. Cytokine measurements were analyzed separately using paired two-tailed t-test. Representative histograms are shown for CXCL10. Panel B was analyzed using repeated-measures two-way ANOVA with treatment and time as factors followed by Šídák’s multiple-comparison test. PD-L1 expression in panel C was analyzed by paired two-tailed t-test. *p < 0.05; **p < 0.01; ***p < 0.001; ****p < 0.0001

Figure S9 IL-8/IL-8-receptor blockade modulates CAF subpopulation distribution in short-term co-culture.

(A) Statistical analysis of CAF subpopulation distribution across control, IL-8–treated, and IL-8 withdrawal conditions. (B) Statistical analysis of only fibroblast subpopulations CD9+ vs CD9- from a TC co-culture for 48h between control, anti-IL8 and anti-IL8 receptor treatment. Data are mean ± SD from n=3 biological replicates. CAF-subpopulation frequencies were analyzed separately using repeated-measures one-way ANOVA followed by Tukey’s multiple-comparison test. “% of parent” indicates the percentage within the gated fibroblast parent population. *p < 0.05; **p < 0.01; ***p < 0.001; ****p < 0.0001

Figure S10 Stromal FAP/FN1 and PD-L1 expression correlate with CD8-positive T-cell infiltration in thyroid carcinoma tissue samples.

(A–B) Immunohistochemical analysis of tissue microarrays (TMA) generated from an in-house TC patient cohort. Correlation of PD-L1 tumor proportion score (TPS) and fibronectin-1 (FN1) expression in connective tissue both converted to H-SCORE with CD8⁺ T cell infiltration. (C) Quantification of CD8⁺ T cells in TMA lesions categorized as FN1 high versus low/negative from the same cohort. Correlation analyses were performed using Spearman correlation. CD8+ T-cell infiltration in FN1-high versus FN1-low/negative lesions was compared using the Mann–Whitney test. *p < 0.05, **p < 0.01, ***p < 0.001, ****p < 0.0001.
